# DNA Sequences Shaped by Selection for Stability

**DOI:** 10.1371/journal.pgen.0020022

**Published:** 2006-02-24

**Authors:** Martin Ackermann, Lin Chao

**Affiliations:** 1 Division of Biological Sciences, University of California San Diego, La Jolla, California, United States of America; 2 ETH Zürich, Theoretical Biology, Zurich, Switzerland; INSERM U571, France

## Abstract

The sequence of a stretch of nucleotides affects its propensity for errors during replication and expression. Are proteins encoded by stable or unstable nucleotide sequences? If selection for variability is prevalent, one could expect an excess of unstable sequences. Alternatively, if selection against targets for errors were substantial, an excess of stable sequences would be expected. We screened the genome sequences of different organisms for an important determinant of stability, the presence of mononucleotide repeats. We find that codons are used to encode proteins in a way that avoids the emergence of mononucleotide repeats, and we can attribute this bias to selection rather than a neutral process. This indicates that selection for stability, rather than for the generation of variation, substantially influences how information is encoded in the genome.

## Synopsis

Mutations are a double-edged sword. Most mutations are deleterious to an organism's fitness. On the other hand, without mutation, evolutionary change cannot occur. The rate of mutation is partially controlled by the organism, and one determinant of the mutation rate is the DNA sequence itself. Some DNA sequences are prone to mutations and errors during gene expression, whereas other sequences are more stable. Do organisms typically use stable or unstable DNA sequences in their genes? Both possibilities might seem plausible, and both have been postulated. To answer this question, the authors studied whether organisms' DNA sequences are more or less stable than expected by chance. Analyzing the genomes of a bacterium, a yeast, and a nematode, they find a overwhelming prevalence of stable DNA sequences, suggesting that selection for genetic stability is more important than selection for the generation of variation.

## Introduction

How faithfully a given stretch of nucleotides is replicated and expressed depends not only on the machinery for DNA and RNA processing in the cell, but also on the sequence of the nucleotide stretch itself. Certain sequences are inherently prone to errors during replication and expression, whereas other sequences are more stable. The stability of a nucleotide sequence can evolve independently of the sequence of the encoded protein. This is a consequence of the redundancy of the genetic code. As 61 codons code for 20 amino acids, any given amino acid sequence can be encoded by different nucleotide sequences that differ in their propensity for errors during replication and expression. Here we ask if the nucleotide sequences actually used by organisms are a random sample of all the possible sequences encoding that particular amino acid sequence, or if they deviate from a random choice in the direction of stability or instability.

It has been speculated that the evolution of unstable sequences could result from selection for novel and advantageous mutations. This idea goes back to reports about high mutation rates in certain loci of pathogenic bacteria [1]. More recently, high local mutation rates have been implied for loci in non-pathogenic bacteria [2] and yeast [3], and it has been speculated that unstable nucleotide sequences could generally make a substantial contribution to genetic variation for selection to act upon [4,5].

The evolution of stable sequences is thought to be the consequence of the costly flipside of instability. Although mutations can occasionally confer benefits, most mutations are deleterious, and a higher mutation rate can lead to a harmful mutational load. Additionally, errors during transcription or translation are metabolically costly. Such combined costs could lead to selection towards alleles with stable nucleotide sequences.

Currently, the relative importance of selection for stability or instability of DNA sequences is not clear. To investigate this question, we asked whether coding sequences of a number of organisms were more or less stable than expected by chance. We focused on one important determinant of stability, the occurrence of mononucleotide repeats: homogenous runs of one nucleotide. Mononucleotide repeats have a strong influence on the local mutation rate [6]. In yeast, extending a mononucleotide repeat (of length ≥4) by one nucleotide leads to an increase in the local mutation rate by about a factor of two [7]. The most common mutations in mononucleotide repeats are insertions or deletions of one or more nucleotide, often leading to a change in the reading frame of the remainder of the protein. This alters the amino acid sequence and typically leads to the emergence of a premature stop codon. Errors during expression are also strongly influenced by mononucleotide repeats: high error rates of transcription [8] and translation [9] have been reported for mononucleotide repeats in Escherichia coli. For this study, we focus on mononucleotide repeats, because repeats consisting of short units are much more common and less stable [10] than repeats of longer units.

The distribution of mononucleotide repeats in organisms' genomes has been addressed by many other studies [11–15]. These studies have often found that the observed number of mononucleotide repeats exceeded the expected number [12,13], and this finding is sometimes interpreted as evidence for selection for evolvability [4,5,16]. A few studies also reported under-representation of mononucleotide repeats [11,14]. The expected number of repeats is usually calculated by assuming that nucleotides or codons are randomly distributed within a gene (but see [17,18]). This null model does not preserve the amino acid sequence of proteins. It would be appropriate if the amino acids were randomly distributed in genes. However, many proteins contain amino acid repeats [19,20] and some of these repeats have functional significance [20]. Amino acid repeats make the emergence of nucleotide repeats more likely and thus increase their numbers relative to a null model that does not take into account the amino acid sequence. This effect could explain the common result that actual nucleotide sequences contain more mononucleotide repeats than the random sequences generated under this particular null model. These studies are thus not sufficient to resolve the question whether the amino acid sequence is encoded in a way that avoids or promotes the emergence of nucleotide repeats.

In contrast to most of these earlier studies, we used a null model that preserves the amino acid sequence. Such null models have been used for comparing observed and expected nucleotide sequences in terms of RNA secondary structure [21], the frequency of short nucleotide motifs in different frames [22], and the frequency of targets for errors during translation [23]. A recent study used such a null model to identify runs of adenines and thymines that are thought to be involved in errors during transcription in bacterial genomes [24]. Here we used this method to investigate the occurrence of mononucleotide repeats in the genomes of *E. coli, Saccharomyces cerevisiae,* and *Caenorhabditis elegans.* We analyzed all genes of these organisms and asked whether they contain more or fewer repeats than expected under this null model. This allowed us to determine whether these organisms use stable or unstable nucleotide sequences to encode their proteins.

## Results/Discussion

### Expected and Observed Number of Repeats

We analyzed the coding regions of all confirmed genes of *C. elegans, S. cerevisiae,* and *E. coli,* for observed and expected number of mononucleotide repeats. One thousand realizations of randomized nucleotide sequences were generated for every gene in each organism. The randomized sequences preserved the amino acid sequence of the genes and the within-gene codon usage frequencies. For each realization, we counted the number and length of all mononucleotide repeats. This allowed for determining the average and variance in the numbers of mononucleotide repeats expected under the null model in which codon usage is independent of the context.

**Figure 1:**
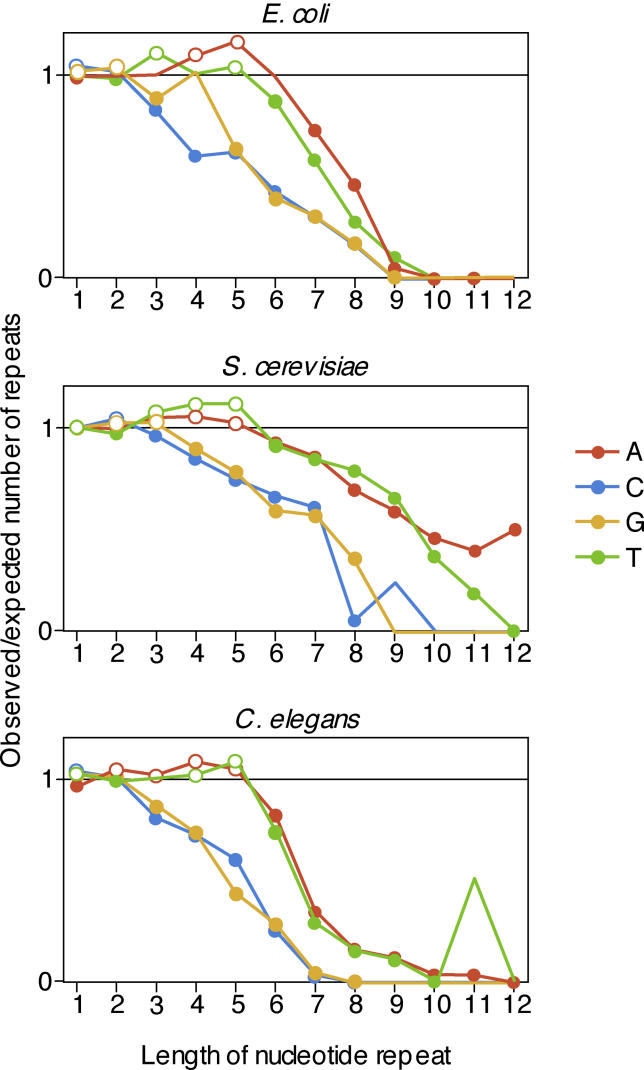
Long Mononucleotide Repeats Are Less Frequent than Expected by Change The figure shows the ratio between observed and expected number of mononucleotide repeats (*y*-axis) as a function of their length (*x*-axis) in all open reading frames of E. coli, S. cerevisiae, and C. elegans. Observed and expected numbers were summed over all the genes in a genome. Each line represents repeats of one nucleotide. While very short repeats occur at about the expected frequencies, longer repeats are consistently rarer than expected. Dots mark cases where the difference between observed and expected number of repeats is significant (at *p* < 0.05, two-sided test, based on 1,000 randomizations). Open dots indicate that the observed number is higher than the expected number; filled dots indicate that the observed number is lower than the expected number.

We then compared the expected numbers of mononucleotide repeats with the numbers observed in the original genome sequences. We found that short repeats occur at about the frequency expected by chance, but longer mononucleotide repeats are substantially rarer than predicted by the null model in all three organisms (Figure 1 and Table S1). The frequencies of long repeats lie outside of the spread generated by repeated randomization, indicating that the deviation does not reflect random fluctuations, but rather a consistent under-representation of long repeats. The bias against repeats is very similar among organisms; it increases with repeat length, and is stronger for repeats of cytosine and guanine than for adenine and thymine. The three organisms differ substantially in GC content (51% in *E. coli,* 39% in *S. cerevisiae,* 36% in C. elegans); that the under-representation of repeats of the different nucleotides is nevertheless very similar suggests that mutational biases are not sufficient to explain the under-representation. Rather, this result is in line with the finding that in many organisms mononucleotide repeats of cytosine and guanine have a higher mutation rate than repeats of adenine and thymine [13,25,26]. If nucleotide repeats are under-represented because they are selected against, one would expect that the more unstable repeats of cytosine and guanine show a stronger under-representation than the more stable repeats of adenine and thymine, as was observed here.

The under-representation of mononucleotide repeats is thus consistent with the hypothesis that coding sequences in these genomes are selected for stability. However, two alternative explanations have to be considered. First, the bias against repeats could result from a neutral process rather than from selection [27,28]. Second, if a neutral process can be ruled out in favor of selection, one has to show that selection is acting against long repeats directly. As will be discussed below, the observed patterns could also be an indirect consequence of other types of selection on codon usage.

### Distinguishing between Neutral Process and Selection

The next question is thus whether the bias against repeats results from selection for stability or from a neutral process. The neutral process that could produce a similar pattern is a context-dependent mutational bias. It is known that the nucleotides adjacent to a site undergoing a mutation can affect the identity of the incoming nucleotide [29]. Such context-dependent mutational biases could, in principle, lead to interruptions of mononucleotide runs with other nucleotides and therefore lead to an under-representation of repeats.

To distinguish between selection and neutral processes, we compared genes that are expected to be under strong selection for stability with genes that are expected to be under weaker selection for stability. If the bias against repeats is stronger in the first group of genes, it can be attributed to selection. The strength of selection against errors during replication should be higher in essential genes, whereas the strength of selection against errors during gene expression should be higher in highly expressed genes, where high error rates lead to large numbers of deficient transcripts of proteins and entail large metabolic costs.

We thus looked for an association between the prevalence of mononucleotide repeats in a gene and its essentiality and expression level. To do so, we derived one single measure summarizing the distribution of repeats of each of the four nucleotides within a gene. This measure is the mean repeat length, calculated by averaging repeat length over all occurrences in an open reading frame of the nucleotide to be investigated, including all cases where this nucleotide occurred alone. We determined the observed average repeat length for every gene and compared it to the average length in randomized sequences that again were generated, while preserving the within-gene codon frequencies and the amino acid sequence. An analysis of covariance was used to test whether the difference between observed and expected average length depended on the expression level of the gene and on its essentiality. For this analysis, genes leading to substantial growth disadvantages or phenotypic changes, when knocked out or knocked down, were included in the list of essential genes.

This analysis showed that in all three organisms, genes that are essential or expressed at high levels tend to have shorter nucleotide runs than genes that are not essential or that are expressed at lower levels (Table S2). In *E. coli,* the effect of expression was significant for the bases A, G, and T. In *S. cerevisiae,* the effect of expression was significant for A and T, and the effect of essentiality was significant for A. In *C. elegans,* the effect of expression was significant for A, C, and T, and the effect of essentiality was significant for A, C, and G (*p* < 0.001 for all significant results; see Table S2 for details). The effects were not strong but consistent: in all significant cases, genes that are essential or expressed at high levels had shorter mononucleotide runs. Importantly, expression level and essentiality did both individually contribute to the bias against repeats. Although essential genes tend to be expressed at higher rates [30] and the two variables are thus associated, the analysis of covariance corrects for this association. Our results thus indicate that selection against repeats is mediated through errors during replication and expression in likewise manners.

In intron sequences, where the errors during replication or expression are inconsequential, no effect of expression, or essentiality on repeat lengths, was observed (analysis with genes of *C. elegans;* unpublished data). This latter finding rules out that transcription-mediated mutational biases [31] were responsible for this result, and strengthens the viewpoint that selection, rather than a neutral process, is responsible for the under-representation of repeats. We would also like to note that the effects of essentiality and expression level were not confounded by codon bias. As mentioned above, randomized sequences were generated for each gene individually while maintaining within-gene codon frequencies. Differences in codon bias between genes that differ in expression levels [32] could thus not influence the analysis.

### Selection Acts Directly against Long Repeats

Having demonstrated that the bias against repeats most likely resulted from selection, we then asked whether selection acts against long repeats directly. Alternatively, the deficiency of long repeats could also be a by-product of selection against sub-units of long repeats. For example, in *E. coli,* the codon TTT is avoided in favor of TTC at positions immediately followed by a T [29]. This reduces the frequency of runs of four thymines, and thus indirectly also of longer stretches of thymine that necessarily contain runs of four.

We tested whether such dependencies of codon choice on the nucleotide immediately following were sufficient to explain our results, or whether we had to invoke direct selection against long repeats. To do so, we asked how the use of codons that can give rise to mononucleotide repeats depended on the sequence context. We again analyzed all coding regions of *C. elegans, S. cerevisiae*, and *E. coli,* and identified all occurrences of the four amino acids that can be encoded by a codon consisting of three identical nucleotides (we call these codons ‘homogenous codon'). These amino acids are phenylalanine, proline, lysine, and glycine. Each of these amino acids can also be encoded by one or more codons that do not consist of three identical nucleotides (‘heterogeneous codons'). We asked whether the choice between homogeneous and heterogeneous codons was altered at positions immediately followed by one or more of the nucleotides constituting the homogeneous codon (in the following, we call these ‘critical nucleotides'). For example, we determined the ratio between TTT and TTC, encoding phenylalanine, as a function of the number of T immediately following. If preference for TTC were mediated through the following nucleotide alone, then one would expect the ratio between TTT and TTC codons to be determined by the nucleotide immediately following, and unaffected by nucleotides further downstream. In contrast, if selection acted directly against long repeats, one would expect that the ratio between TTT and TTC codons would be continuously lowered at positions followed by runs of T of increasing length. This is because TTT at such positions gives rise to long repeats.

**Figure 2:**
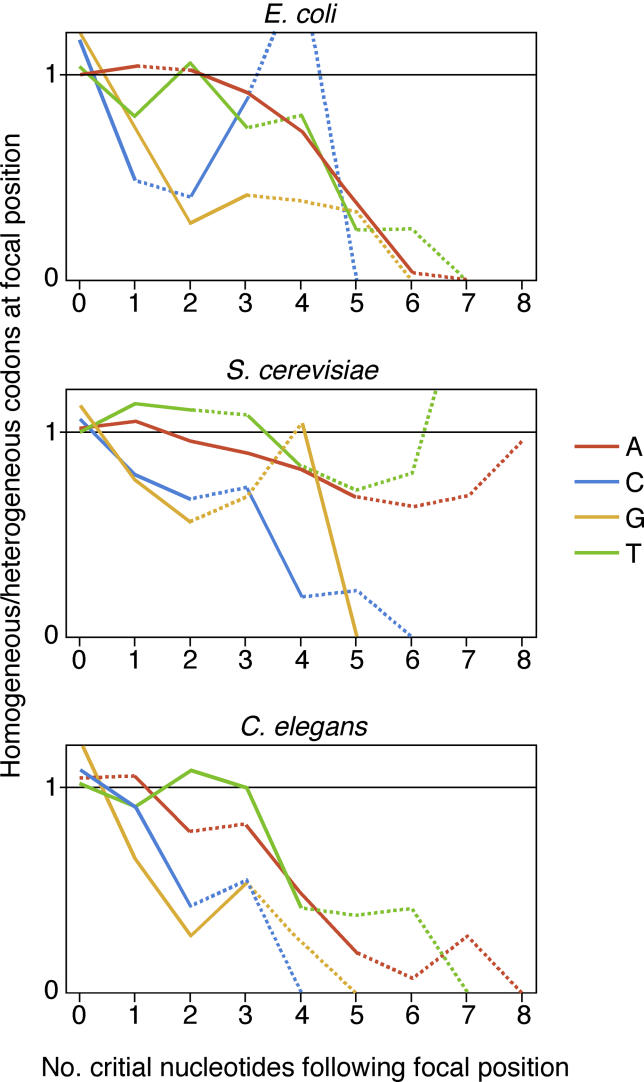
The Bias against Mononucleotide Repeats Is a Consequence of Context-Dependent Codon Choice We analyzed codons choice for the four amino that can be encoded by a homogenous codon (NNN, N ∈ {A, C, G, T}) as well as by one or more heterogeneous codons (NNX, X ≠ N). The ratio of homogeneous and heterogeneous codons encoding these amino acids (*y*-axis) is plotted in dependence of the number of nucleotides constituting the homogeneous codon (N) immediately following (*x*-axis). This ratio decreases at positions followed by runs of the critical nucleotide (N) of increasing length. The figure is based on all occurrences of these four amino acids from open reading frames of E. coli, S. cerevisiae, and C. elegans. Each line represents one nucleotide. The *y*-axis is standardized by the genome-wide average ratio between homogeneous and heterogeneous codons for that particular nucleotide. Solid lines between x = i and x = i+1 indicate that the ratio between homogeneous codon and heterogeneous codons differs significantly between positions followed by i and i+1 of the critical nucleotide (*p* < 0.05, chi-square test). Dotted lines indicate that the difference is not significant.

We found this latter scenario to be true: the ratio between homogeneous and heterogeneous codons was decreased at positions followed by runs of increasing length of the critical nucleotide (Figure 2). The context influencing codon choice was quite large; for example, while homogeneous codons were generally avoided at positions followed by four of the critical nucleotides in a row, their frequency was further decreased when a fifth critical nucleotide was added to the run of four. In five out of 12 cases (each combination of organism and nucleotide representing one case), this decrease was significant (chi-square test, Figure 2). The effect of the immediately following nucleotide was not only insufficient to account for this discrimination against homogeneous codons, but sometimes even acted in the opposite direction. In four out of 12 cases (again, each combination of organism and nucleotide representing one case), having one instead of zero critical nucleotides immediately following increased the chance for a homogenous codon.

As a second test for how codon choice depended on the sequence context, we used logistic regression; we investigated how the ratio between homogeneous and heterogeneous codons related to the number of critical codons immediately following. Whereas the chi-square test described in Figure 2 tested how this ratio changed with each increment of one critical nucleotide following the focal codon, the logistic regression tested for an overall trend in how the ratio between homogeneous and heterogeneous codons changed if followed by runs of the critical nucleotide of increasing lengths. This analysis showed that in eleven out of 12 cases (each combination of organism and nucleotide representing one case), the probability for a homogeneous codon decreased significantly (at *p* <0.01) with increasing numbers of critical nucleotides following (Table S3). In one case (for the base T in S. cerevisiae), the probability for using a homogeneous codon increased with increasing numbers of critical nucleotides following. These results strongly suggest that the bias against long nucleotide repeats is due to selection acting specifically against them.

### Investigating the Source of Selection against Repeats

The source of this selection against repeats could be any of the processes whose stability is affected by the presence of nucleotide repeats—DNA replication, transcription, or translation. One can gain insights on the relative importance of these three processes by analyzing repeats that are interrupted by introns. Such repeats do not affect the stability of DNA replication or transcription. They emerge after splicing and potentially interfere with the stability of translation. For this reason, only selection for translational stability can lead to a bias against repeats that span introns. We analyzed coding sequences in C. elegans and identified positions that potentially lead to the emergence of nucleotide repeats interrupted by introns. Such positions are frequent for repeats of A, but not for repeats of the other nucleotides; we thus focused on the former in order to get sufficient sample size.

Specifically, we identified positions where lysine (encoded by AAA or AAG) was immediately followed by an intron. Two cases were distinguished: in the first, there was a stretch of at least three A immediately following the intron. In the second, there were fewer than three A (often zero) following the intron. In the first case (but not in the second), encoding the lysine preceding the intron with AAA leads to a repeat of six A or more spanning the intron. If stability during translation is an important force selecting against repeats, such sequences should be avoided. However, this was not the case. Lysines that precede introns are encoded by a AAA in about 20% of the cases irrespective of whether the intron is followed by a stretch of A or not (19.2% if followed by at least three A; 21.9% if followed by less than three A; chi-square = 2.575, *n* = 11,035, *p* = 0.1136). This contrasts with codon choice for lysines that are not followed by introns. There, AAA is used in 61% of the cases at positions followed by less than three A, but at only 45% at positions followed by three or more A (chi-square = 2,743, *n* = 605,696, *p* < 0.0001).

The interpretation of this result is complicated by the fact that exonic sequences at splice sites differ from other positions in terms of nucleotide frequencies. For example, the consensus sequence for 3′ ends of exons in C. elegans is AG [33], which explains why lysines preceding introns are predominantly encoded by AAG rather than AAA. Our analysis thus hinges on the assumption that the sequence at the 3′ end of an exon does not interact with the sequence at the 5′ end of the next exon (such interactions could lead to a bias for or against repeats spanning introns). Under this assumption, the fact that repeats that are interrupted by introns are not under-represented suggests that stability during translation is not an important source of selection against repeats.

What can we thus conclude on the sources of selection against repeats? There are three different sources: stability of replication, transcription, and translation. The analysis of covariance suggested that stability during replication and expression both contributed to the selection against repeats, indicating that replication was not the only important factor. The analysis of the influence of the sequence context on codon choice showed that selection acted directly against long repeats, and that codon choice that lead to the bias against repeats was not mainly driven by the nucleotide immediately following. Such distant effects are probably not indicative of selection for translational stability, and might rather suggest replication or transcription as the source of selection for stability. The analysis of repeats spanning introns also did not support translational stability as an important source of selection against repeats. Taken together the results thus suggest that the selection against repeats results from the stability of replication and transcription, whereas there is no evidence for an effect of translation.

The bias against repeats reported here stands in contrast to earlier studies that found an excess of mononucleotide repeats in the same organisms [12,13]. These previous findings were based on models that did not preserve the amino acid sequence, and the difference between our results and those results are a consequence of the different null model. Null models that do preserve the amino acid sequence have also been used before to investigate the distribution of mononucleotide repeats, and these studies reported cases of under-representation of certain types of repeats [17,18,24]. Our study corroborates and extends these results; our results suggest that the bias against repeats is a general pattern for all four nucleotides and across different organisms, and that the bias is a consequence of selection for stability.

These results do not preclude that some genes, mostly in pathogenic bacteria, indeed contain repeats as a mechanism to promote variation. This assertion is supported by compelling evidence [1,34]. However, we conclude that such cases are exceptions, and that the majority of genes are selected for stability against errors during replication and expression.

## Materials and Methods

### Origin of data.

Sequence data of E. coli K12 was retrieved from NCBI. Expression data was obtained from [35]. The five experiments with the wild-type from the calibrated microarray experiments (ID = EXPSET0003) were analyzed. The datasets were log-transformed and standardized by subtracting the mean and dividing by the standard deviation. For each open reading frame (ORF), the average over these five standardized datasets was used as the mean expression level. Essentiality data was obtained from [36]. These data derive from a transposon-insertion study, a method that labels genes as essential if their inactivation is lethal or leads to a substantial growth disadvantage.

Sequence data of S. cerevisiae was retrieved from the *Saccharomyces* Genome Database (http://www.yeastgenome.org, accessed June 9 2004). Expression data was obtained from [37]. The data from time point 0 was retrieved from each of the seven experiments; the seven datasets were log-transformed and normalized as above. For each ORF, the average over the seven standardized datasets was used as the mean expression level. Essentiality data was retrieved from two sources: the first source is the list of essential genes from the *Saccharomyces* Genome Database (http://www.yeastgenome.org, accessed 9 June 2004), and the second source is a list of growth rate measures with knockout strains of yeast from [38]. Genes with growth rates lower than 0.95 were added to the list of essential genes.

Sequence data of C. elegans was retrieved from WormBase (http://www.wormbase.org) release WS123 (confirmed genes). Expression data of C. elegans was obtained from [39]. The average expression level was defined as the logarithm of the arithmetic mean over all life-stages. Essentiality data was obtained from [40]. This dataset lists all ORFs that produce substantial phenotypic alterations when knocked-down by RNAi.

### Determining the expected distribution of mononucleotide repeats lengths.

We used randomization to determine the distribution of mononucleotide repeats under the null model that codons were used according to their ORF-specific frequencies, but independent of the context. The codon frequencies within each ORF of each of the three organisms were determined, and 1,000 random rearrangements of the each ORF's nucleotide sequence were generated that preserved the amino acid sequence and that were based on the within-ORF codon frequencies. For randomization, codons were drawn according to their within-ORF frequencies, but with replacement; the randomized sequences thus typically differed slightly from the observed sequences in their codon frequencies.

In these randomized sequences as well as in the original sequence the number and length of all mononucleotide repeats of length ≥1 was determined. PERL programs written by the authors were used to determine of the codon frequencies, for randomizations, and to count the number of repeats.

### Statistical analysis.

To determine the whether the observed distribution of mononucleotide repeats deviated from the null-expectation, we compared it to the distributions derived from the 1,000 randomized sequences generated under the null model. For each combination of nucleotide (A, C, G, and T) and mononucleotide repeat length (ranging from one to 12, where one refers to the single occurrence of a nucleotide), we compared the observed number of observations to the 1,000 numbers generated under the null model (observed and expected numbers of repeats of a given nucleotide and length were summed over all the genes within a genome). A significant deviation between observation and expectation was concluded if the observed number did not fall in a percentile range of 2.5–97.5 of the distribution generated by randomization.

To test for an association between repeat length in a gene and its expression level and essentiality, we determined for each ORF of each of the three organisms the observed and expected mean length of mononucleotide repeats of A, C, G, and T. To determine the observed length, we listed all mononucleotide of the nucleotide to be investigated (ranging in length from 1 for single occurrences of the nucleotide to the length of the longest observed run) within a gene and averaged these values to obtain the mean length. To determine the expected mean length, we created ten randomized sequences for each gene (preserving the amino acid sequence and the within-gene codon frequencies). For each randomized gene sequence, we again determined the mean length for repeats of each nucleotide, and averaged the mean length over the ten randomized sequences. Then, we determined the fraction by which the observed mean length exceeded the expected mean length, for every gene and every nucleotide (this fraction is equal to (observed length-expected length)/expected length). This quantity was used as the response variable in an analysis of covariance with the fixed factor essentiality (with the two levels ‘essential' and ‘non-essential') and the covariable expression level. The program JMP (version 5.1, SAS Institute, Cary, North Carolina, United States) was used for the analysis of covariance. The same analysis was also carried out with intron sequences from *C. elegans.*

To determine the effect of the context on the ratios of homogeneous to heterogeneous codons, we proceeded as follows. In all ORFs of each of the three organisms we determined all occurrences of the four amino acids that can be encoded by a homogeneous codon (three times the same nucleotide) as well as one or more heterogeneous codons (not three times the same nucleotide). These are phenylalanine (TTT and TTC), proline (CCA, CCC, CCG, and CCT), lysine (AAA and AAC), and glycine (GGA, GGC, GGG, and GGT). For each occurrence of one of these codons, we asked whether the codon was immediately followed by one or more of the nucleotides constituting the respective homogeneous codon, and if yes, by how many. This number ranged from zero to the maximal observed value, 13. This allowed determining the ratio between homogeneous and heterogeneous codons as a function of the number of nucleotides constituting the homogeneous codons immediately following. As this test is based on every occurrence of these codons, a single mononucleotide repeat will produce more than one observation; each codon within a repeat counted as one observation.

We used two different statistical methods to analyze whether the ratio between homogeneous and heterogeneous codons depended on the number of critical nucleotides following. First, a chi-square test was used to compare the ratio of homogeneous to heterogeneous codons between every pair of i and i+1 (i = 1–12) critical nucleotides immediately following. Second, logistic regression was used to test whether the probability for a homogeneous codon changed with the number of critical nucleotides following. The program JMP (version 5.1, SAS Institute) was used for logistic regression.

## Supporting Information

Table S1 Observed and Expected Number of Mononucleotide Repeats in Coding Regions of the Genomes of E. coli, S. cerevisiae, and C. elegans
The expected numbers are based on 1,000 randomizations that preserved amino-acid sequence and within-gene codon frequencies. The arithmetic mean and the 2.5th and the 97.5th percentile of the expected numbers are reported.
(274 KB DOC)

Table S2 Results of the Analysis of Covariance The response variable is the relative difference between observed and expected repeats length [(observed-expected)/expected]. The explanatory variables are the binary variable essentiality and the covariate expression.
(258 KB DOC)

Table S3 Results of the Logistic Regression The binary response variable is codon choice (heterogeneous codon vs. homogeneous codon); the explanatory variable is the number of critical nucleotides following. In 11 out of 12 tests, the probability for a heterogeneous codon increased significantly with increasing numbers of critical nucleotides following.
(187 KB DOC)

### Accession Numbers

The National Center for Biotechnology Information (NCBI) (http://www.ncbi.nlm.nih.gov) accession number for E. coli K12 is 000913.

## Abbreviation

ORF: open reading frame
