# Supplementary material for: DNA Sequences Shaped by Selection for Stability
Source: PLoS Genet. 2006 Feb 24;2(2):e22. doi: 10.1371/journal.pgen.0020022 (PMC1378130; doi:10.1371/journal.pgen.0020022)
Supplement: Table S1 — The expected numbers are based on 1,000 randomizations that preserved amino-acid sequence and within-gene codon frequencies. The arithmetic mean and the 2.5th and the 97.5th percentile of the expected numbers are reported. (274 KB DOC) [file pgen.0020022.st001.doc]

Table S1: Observed and Expected Number of Mononucleotide Repeats (with 2.5 and 97.5 Percentile from 1000 Randomizations)

| ***E. coli*** | |  |  |  |  |  |  |  |  |  |  |  |  |  |  |  |  |  |  |
| --- | --- | --- | --- | --- | --- | --- | --- | --- | --- | --- | --- | --- | --- | --- | --- | --- | --- | --- | --- |
|  | A |  |  |  |  | C |  |  |  |  | G |  |  |  |  | T |  |  |  |
| Len | Observed | Expected | 2.5 PCTL | 97.5 PCTL |  | Observed | Expected | 2.5 PCTL | 97.5 PCTL |  | Observed | Expected | 2.5 PCTL | 97.5 PCTL |  | Observed | Expected | 2.5 PCTL | 97.5 PCTL |
| 1 | 479679 | 486302 | 485516 | 487045 |  | 607157 | 583473 | 582468 | 584438 |  | 619905 | 612620 | 611739 | 613419 |  | 531299 | 535345 | 534398 | 536220 |
| 2 | 133515 | 133723 | 133276 | 134194 |  | 150828 | 148994 | 148482 | 149582 |  | 185052 | 180014 | 179511 | 180510 |  | 133886 | 136827 | 136303 | 137358 |
| 3 | 45113 | 45159 | 44897 | 45422 |  | 25839 | 31345 | 31066 | 31607 |  | 30719 | 34796 | 34551 | 35044 |  | 40291 | 36374 | 36083 | 36679 |
| 4 | 14646 | 13310 | 13151 | 13466 |  | 3224 | 5353 | 5227 | 5484 |  | 7022 | 6976 | 6830 | 7115 |  | 10546 | 10560 | 10400 | 10727 |
| 5 | 6095 | 5227 | 5135 | 5331 |  | 496 | 811 | 754 | 864 |  | 1198 | 1838 | 1755 | 1917 |  | 3149 | 3030 | 2936 | 3130 |
| 6 | 1967 | 1980 | 1919 | 2041 |  | 68 | 157 | 134 | 181 |  | 151 | 392 | 358 | 429 |  | 808 | 938 | 889 | 993 |
| 7 | 375 | 521 | 485 | 554 |  | 9 | 29 | 19 | 40 |  | 28 | 90 | 72 | 108 |  | 170 | 288 | 259 | 319 |
| 8 | 76 | 162 | 143 | 183 |  | 1 | 6.2 | 2 | 12 |  | 4 | 25 | 17 | 36 |  | 22 | 82 | 66 | 98 |
| 9 | 3 | 56 | 45 | 68 |  | 0 | 1.4 | 0 | 4 |  | 0 | 6 | 2 | 11 |  | 2 | 23 | 15 | 31 |
| 10 | 0 | 15 | 9 | 22 |  | 0 | 0.4 | 0 | 2 |  | 0 | 1.3 | 0 | 4 |  | 0 | 5.7 | 2 | 10 |
| 11 | 0 | 4 | 1 | 7 |  | 0 | 0.1 | 0 | 1 |  | 0 | 0.5 | 0 | 2 |  | 0 | 1.9 | 0 | 5 |
| 12 | 0 | 1.2 | 0 | 3 |  | 0 | 0 | 0 | 0 |  | 0 | 0.1 | 0 | 1 |  | 0 | 0.6 | 0 | 2 |
| 13 | 0 | 1.2 | 0 | 3 |  | 0 | 0 | 0 | 0 |  | 0 | 0 | 0 | 1 |  | 0 | 0.3 | 0 | 2 |
| 14 | 0 | 0.2 | 0 | 1 |  | 0 | 0 | 0 | 0 |  | 0 | 0 | 0 | 0 |  | 0 | 0 | 0 | 0 |
| 15 | 0 | 0 | 0 | 0 |  | 0 | 0 | 0 | 0 |  | 0 | 0 | 0 | 0 |  | 0 | 0.1 | 0 | 1 |
| 16 | 0 | 0 | 0 | 0 |  | 0 | 0 | 0 | 0 |  | 0 | 0 | 0 | 0 |  | 0 | 0 | 0 | 0 |
| 17 | 0 | 0 | 0 | 0 |  | 0 | 0 | 0 | 0 |  | 0 | 0 | 0 | 0 |  | 0 | 0 | 0 | 0 |
| 18 | 0 | 0 | 0 | 0 |  | 0 | 0 | 0 | 0 |  | 0 | 0 | 0 | 0 |  | 0 | 0 | 0 | 0 |
|  |  |  |  |  |  |  |  |  |  |  |  |  |  |  |  |  |  |  |  |
| ***S. cerevisiae*** | |  |  |  |  |  |  |  |  |  |  |  |  |  |  |  |  |  |  |
|  | A |  |  |  |  | C |  |  |  |  | G |  |  |  |  | T |  |  |  |
| Len | Observed | Expected | 2.5 PCTL | 97.5 PCTL |  | Observed | Expected | 2.5 PCTL | 97.5 PCTL |  | Observed | Expected | 2.5 PCTL | 97.5 PCTL |  | Observed | Expected | 2.5 PCTL | 97.5 PCTL |
| 1 | 874898 | 884729 | 883546 | 885805 |  | 780672 | 781160 | 780028 | 782188 |  | 825323 | 830464 | 829502 | 831443 |  | 833625 | 842463 | 841266 | 843680 |
| 2 | 327824 | 331086 | 330345 | 331822 |  | 178179 | 172330 | 171718 | 172980 |  | 203140 | 198962 | 198439 | 199472 |  | 271840 | 282870 | 282128 | 283582 |
| 3 | 113345 | 107901 | 107459 | 108353 |  | 29013 | 30525 | 30212 | 30825 |  | 33250 | 32717 | 32467 | 32976 |  | 90538 | 84352 | 83939 | 84756 |
| 4 | 41812 | 39791 | 39504 | 40066 |  | 5493 | 6526 | 6382 | 6676 |  | 5157 | 5816 | 5684 | 5952 |  | 28592 | 25741 | 25471 | 25990 |
| 5 | 14824 | 14624 | 14442 | 14810 |  | 948 | 1283 | 1219 | 1351 |  | 931 | 1201 | 1139 | 1266 |  | 8101 | 7319 | 7184 | 7451 |
| 6 | 4886 | 5352 | 5237 | 5464 |  | 160 | 242 | 212 | 274 |  | 133 | 225 | 196 | 253 |  | 2093 | 2335 | 2255 | 2419 |
| 7 | 1704 | 2010 | 1941 | 2086 |  | 35 | 58 | 43 | 73 |  | 28 | 49 | 37 | 63 |  | 584 | 694 | 650 | 741 |
| 8 | 528 | 770 | 723 | 817 |  | 1 | 18 | 10 | 28 |  | 4 | 11 | 5 | 19 |  | 153 | 196 | 173 | 220 |
| 9 | 161 | 275 | 246 | 304 |  | 1 | 4.1 | 1 | 8 |  | 0 | 2.5 | 0 | 6 |  | 40 | 62 | 47 | 75 |
| 10 | 48 | 107 | 89 | 126 |  | 0 | 1.2 | 0 | 4 |  | 0 | 0.5 | 0 | 2 |  | 7 | 20 | 12 | 29 |
| 11 | 20 | 51 | 39 | 64 |  | 0 | 0.7 | 0 | 3 |  | 0 | 0.2 | 0 | 1 |  | 1 | 5.6 | 2 | 11 |
| 12 | 8 | 16 | 9 | 24 |  | 0 | 0.1 | 0 | 1 |  | 0 | 0 | 0 | 1 |  | 0 | 1.8 | 0 | 4 |
| 13 | 0 | 7.4 | 3 | 13 |  | 0 | 0.1 | 0 | 1 |  | 0 | 0 | 0 | 0 |  | 0 | 0.6 | 0 | 3 |
| 14 | 2 | 5.7 | 2 | 10 |  | 0 | 0 | 0 | 1 |  | 0 | 0 | 0 | 0 |  | 0 | 0.2 | 0 | 1 |
| 15 | 1 | 2.4 | 0 | 6 |  | 0 | 0 | 0 | 0 |  | 0 | 0 | 0 | 0 |  | 0 | 0.1 | 0 | 1 |
| 16 | 1 | 1.1 | 0 | 4 |  | 0 | 0 | 0 | 0 |  | 0 | 0 | 0 | 0 |  | 0 | 0 | 0 | 0 |
| 17 | 0 | 1 | 0 | 3 |  | 0 | 0 | 0 | 0 |  | 0 | 0 | 0 | 0 |  | 0 | 0 | 0 | 0 |
| 18 | 0 | 0.3 | 0 | 1 |  | 0 | 0 | 0 | 0 |  | 0 | 0 | 0 | 0 |  | 0 | 0 | 0 | 0 |
|  |  |  |  |  |  |  |  |  |  |  |  |  |  |  |  |  |  |  |  |
| ***C. elegans*** | |  |  |  |  |  |  |  |  |  |  |  |  |  |  |  |  |  |  |
|  | A |  |  |  |  | C |  |  |  |  | G |  |  |  |  | T |  |  |  |
| Len | Observed | Expected | 2.5 PCTL | 97.5 PCTL |  | Observed | Expected | 2.5 PCTL | 97.5 PCTL |  | Observed | Expected | 2.5 PCTL | 97.5 PCTL |  | Observed | Expected | 2.5 PCTL | 97.5 PCTL |
| 1 | 754558 | 779920 | 779008 | 780900 |  | 814365 | 786572 | 785512 | 787661 |  | 817101 | 804026 | 803157 | 804949 |  | 732159 | 724496 | 723405 | 725576 |
| 2 | 273625 | 261696 | 261060 | 262342 |  | 184369 | 184805 | 184194 | 185411 |  | 207708 | 205168 | 204705 | 205676 |  | 228471 | 231752 | 231097 | 232457 |
| 3 | 78169 | 76474 | 76075 | 76848 |  | 24589 | 30518 | 30217 | 30799 |  | 24783 | 28485 | 28266 | 28722 |  | 53903 | 53932 | 53575 | 54289 |
| 4 | 28951 | 26621 | 26374 | 26860 |  | 4217 | 5846 | 5715 | 5973 |  | 2884 | 3902 | 3799 | 4004 |  | 16120 | 15778 | 15561 | 15998 |
| 5 | 10492 | 10030 | 9872 | 10201 |  | 484 | 810 | 754 | 862 |  | 335 | 761 | 705 | 814 |  | 4471 | 4128 | 4010 | 4238 |
| 6 | 2896 | 3546 | 3453 | 3640 |  | 35 | 141 | 119 | 163 |  | 38 | 135 | 111 | 158 |  | 806 | 1105 | 1045 | 1164 |
| 7 | 411 | 1223 | 1164 | 1283 |  | 1 | 36 | 24 | 48 |  | 1 | 26 | 18 | 36 |  | 90 | 315 | 282 | 347 |
| 8 | 74 | 468 | 432 | 503 |  | 0 | 11 | 5 | 18 |  | 0 | 8.2 | 3 | 14 |  | 11 | 75 | 59 | 91 |
| 9 | 19 | 157 | 135 | 178 |  | 0 | 2.3 | 0 | 6 |  | 0 | 1.4 | 0 | 4 |  | 2 | 19 | 11 | 27 |
| 10 | 2 | 57 | 44 | 71 |  | 0 | 0.7 | 0 | 3 |  | 0 | 0.2 | 0 | 1 |  | 0 | 6.7 | 2 | 12 |
| 11 | 1 | 31 | 21 | 41 |  | 0 | 0.3 | 0 | 2 |  | 0 | 0.2 | 0 | 1 |  | 1 | 2 | 0 | 5 |
| 12 | 0 | 12 | 7 | 19 |  | 0 | 0 | 0 | 1 |  | 0 | 0.1 | 0 | 1 |  | 0 | 0.5 | 0 | 2 |
| 13 | 0 | 5.4 | 1 | 10 |  | 0 | 0 | 0 | 0 |  | 0 | 0 | 0 | 0 |  | 0 | 0.3 | 0 | 2 |
| 14 | 0 | 3.9 | 1 | 8 |  | 0 | 0 | 0 | 0 |  | 0 | 0 | 0 | 0 |  | 0 | 0.2 | 0 | 1 |
| 15 | 0 | 2 | 0 | 5 |  | 0 | 0 | 0 | 0 |  | 0 | 0 | 0 | 0 |  | 0 | 0.1 | 0 | 1 |
| 16 | 0 | 0.7 | 0 | 2 |  | 0 | 0 | 0 | 0 |  | 0 | 0 | 0 | 0 |  | 0 | 0 | 0 | 0 |
| 17 | 0 | 0.6 | 0 | 2 |  | 0 | 0 | 0 | 0 |  | 0 | 0 | 0 | 0 |  | 0 | 0 | 0 | 0 |
| 18 | 0 | 0.2 | 0 | 1 |  | 0 | 0 | 0 | 0 |  | 0 | 0 | 0 | 0 |  | 0 | 0 | 0 | 0 |

Abbreviations: Nuc: Nucleotide; Len: Length; Obs: Observed; Exp: Expected; PCTL: Percentile
