# Supplementary material for: DNA Sequences Shaped by Selection for Stability
Source: PLoS Genet. 2006 Feb 24;2(2):e22. doi: 10.1371/journal.pgen.0020022 (PMC1378130; doi:10.1371/journal.pgen.0020022)
Supplement: Table S2 — The response variable is the relative difference between observed and expected repeats length [(observed-expected)/expected]. The explanatory variables are the binary variable essentiality and the covariate expression. (258 KB DOC) [file pgen.0020022.st002.doc]

Table S2: Analysis of Covariance

| ***E. coli*** | |  |  |  |  |  |  |  |  |  |  |  |  |  |  |
| --- | --- | --- | --- | --- | --- | --- | --- | --- | --- | --- | --- | --- | --- | --- | --- |
|  |  |  |  | Analysis of Variance | |  |  |  |  |  | Parameter Estimates | |  |  |  |
| Nuc | Obs | R2 |  | Source | DF | Sum of Squares | Mean Square | F Ratio | Prob > F |  | Term | Estimate | Std Error | t Ratio | Prob>|t| |
| A | 3646 | 0.009 |  | Model | 3 | 0.015 | 0.0050 | 11.63 | <.0001 |  | Intercept | 0.00719 | 0.00050 | 14.5 | <.0001 |
|  |  |  |  | Error | 3642 | 1.563 | 0.0004 |  |  |  | exp | -0.00219 | 0.00046 | -4.73 | <.0001 |
|  |  |  |  | C. Total | 3645 | 1.578 | 0.0000 |  |  |  | ess[0] | 0.00048 | 0.00049 | 0.98 | 0.3285 |
|  |  |  |  |  |  |  |  |  |  |  | (exp-0.01928)*ess[0] | 0.00031 | 0.00046 | 0.67 | 0.5009 |
|  |  |  |  |  |  |  |  |  |  |  |  |  |  |  |  |
| C | 3646 | 0.002 |  | Model | 3 | 0.004 | 0.0014 | 3.04 | 0.0278 |  | Intercept | -0.02026 | 0.00052 | -39.17 | <.0001 |
|  |  |  |  | Error | 3642 | 1.702 | 0.0005 |  |  |  | exp | 0.00021 | 0.00048 | 0.44 | 0.6626 |
|  |  |  |  | C. Total | 3645 | 1.707 | 0.0000 |  |  |  | ess[0] | -0.00065 | 0.00051 | -1.26 | 0.2064 |
|  |  |  |  |  |  |  |  |  |  |  | (exp-0.01928)*ess[0] | -0.00104 | 0.00048 | -2.16 | 0.0308 |
|  |  |  |  |  |  |  |  |  |  |  |  |  |  |  |  |
| G | 3646 | 0.051 |  | Model | 3 | 0.075 | 0.0251 | 64.58 | <.0001 |  | Intercept | -0.00694 | 0.00047 | -14.72 | <.0001 |
|  |  |  |  | Error | 3642 | 1.413 | 0.0004 |  |  |  | exp | -0.00549 | 0.00044 | -12.48 | <.0001 |
|  |  |  |  | C. Total | 3645 | 1.488 | 0.0000 |  |  |  | ess[0] | -0.00034 | 0.00047 | -0.73 | 0.4661 |
|  |  |  |  |  |  |  |  |  |  |  | (exp-0.01928)*ess[0] | 0.00133 | 0.00044 | 3.02 | 0.0026 |
|  |  |  |  |  |  |  |  |  |  |  |  |  |  |  |  |
| T | 3646 | 0.003 |  | Model | 3 | 0.005 | 0.0016 | 3.37 | 0.0178 |  | Intercept | 0.00515 | 0.00052 | 9.97 | <.0001 |
|  |  |  |  | Error | 3642 | 1.700 | 0.0005 |  |  |  | exp | -0.00134 | 0.00048 | -2.78 | 0.0055 |
|  |  |  |  | C. Total | 3645 | 1.704 | 0.0000 |  |  |  | ess[0] | 0.00027 | 0.00051 | 0.53 | 0.5966 |
|  |  |  |  |  |  |  |  |  |  |  | (exp-0.01928)*ess[0] | 0.00069 | 0.00048 | 1.43 | 0.1515 |
|  |  |  |  |  |  |  |  |  |  |  |  |  |  |  |  |
| ***S. cerevisiae*** | |  |  |  |  |  |  |  |  |  |  |  |  |  |  |
|  |  |  |  | Analysis of Variance | |  |  |  |  |  | Parameter Estimates | |  |  |  |
| Nuc | Obs | R2 |  | Source | DF | Sum of Squares | Mean Square | F Ratio | Prob > F |  | Term | Estimate | Std Error | t Ratio | Prob>|t| |
| A | 5449 | 0.015 |  | Model | 3 | 0.033 | 0.0109 | 27.58 | <.0001 |  | Intercept | 0.00390 | 0.00029 | 13.55 | <.0001 |
|  |  |  |  | Error | 5445 | 2.149 | 0.0004 |  |  |  | exp | -0.00225 | 0.00030 | -7.57 | <.0001 |
|  |  |  |  | C. Total | 5448 | 2.181 | 0.0000 |  |  |  | ess[0] | 0.00076 | 0.00029 | 2.65 | 0.008 |
|  |  |  |  |  |  |  |  |  |  |  | (exp-0.02176)*ess[0] | -0.00015 | 0.00030 | -0.5 | 0.6181 |
|  |  |  |  |  |  |  |  |  |  |  |  |  |  |  |  |
| C | 5449 | 0.001 |  | Model | 3 | 0.003 | 0.0010 | 2.60 | 0.0501 |  | Intercept | -0.00276 | 0.00028 | -9.69 | <.0001 |
|  |  |  |  | Error | 5445 | 2.100 | 0.0004 |  |  |  | exp | 0.00009 | 0.00029 | 0.29 | 0.7708 |
|  |  |  |  | C. Total | 5448 | 2.103 | 0.0000 |  |  |  | ess[0] | 0.00018 | 0.00028 | 0.64 | 0.5201 |
|  |  |  |  |  |  |  |  |  |  |  | (exp-0.02176)*ess[0] | -0.00081 | 0.00029 | -2.75 | 0.006 |
|  |  |  |  |  |  |  |  |  |  |  |  |  |  |  |  |
| G | 5449 | 0.000 |  | Model | 3 | 0.001 | 0.0003 | 0.71 | 0.5455 |  | Intercept | 0.00083 | 0.00027 | 3.05 | 0.0023 |
|  |  |  |  | Error | 5445 | 1.940 | 0.0004 |  |  |  | exp | 0.00005 | 0.00028 | 0.17 | 0.8685 |
|  |  |  |  | C. Total | 5448 | 1.940 | 0.0000 |  |  |  | ess[0] | 0.00017 | 0.00027 | 0.64 | 0.5239 |
|  |  |  |  |  |  |  |  |  |  |  | (exp-0.02176)*ess[0] | 0.00034 | 0.00028 | 1.21 | 0.2252 |
|  |  |  |  |  |  |  |  |  |  |  |  |  |  |  |  |
| T | 5449 | 0.014 |  | Model | 3 | 0.034 | 0.0112 | 25.24 | <.0001 |  | Intercept | 0.00805 | 0.00031 | 26.37 | <.0001 |
|  |  |  |  | Error | 5445 | 2.422 | 0.0004 |  |  |  | exp | -0.00272 | 0.00032 | -8.65 | <.0001 |
|  |  |  |  | C. Total | 5448 | 2.455 | 0.0000 |  |  |  | ess[0] | -0.00040 | 0.00031 | -1.33 | 0.185 |
|  |  |  |  |  |  |  |  |  |  |  | (exp-0.02176)*ess[0] | 0.00041 | 0.00032 | 1.31 | 0.1894 |
|  |  |  |  |  |  |  |  |  |  |  |  |  |  |  |  |
| ***C. elegans*** | |  |  |  |  |  |  |  |  |  |  |  |  |  |  |
|  |  |  |  | Analysis of Variance | |  |  |  |  |  | Parameter Estimates | |  |  |  |
| Nuc | Obs | R2 |  | Source | DF | Sum of Squares | Mean Square | F Ratio | Prob > F |  | Term | Estimate | Std Error | t Ratio | Prob>|t| |
| A | 6861 | 0.037 |  | Model | 3 | 0.112 | 0.0373 | 87.39 | <.0001 |  | Intercept | 0.01801 | 0.00080 | 22.54 | <.0001 |
|  |  |  |  | Error | 6857 | 2.928 | 0.0004 |  |  |  | exp | -0.00871 | 0.00078 | -11.11 | <.0001 |
|  |  |  |  | C. Total | 6860 | 3.040 | 0.0000 |  |  |  | ess[0] | 0.00144 | 0.00041 | 3.47 | 0.0005 |
|  |  |  |  |  |  |  |  |  |  |  | (exp-0.66943)*ess[0] | 0.00107 | 0.00078 | 1.37 | 0.1715 |
|  |  |  |  |  |  |  |  |  |  |  |  |  |  |  |  |
| C | 6861 | 0.021 |  | Model | 3 | 0.058 | 0.0194 | 47.95 | <.0001 |  | Intercept | -0.01535 | 0.00078 | -19.71 | <.0001 |
|  |  |  |  | Error | 6857 | 2.781 | 0.0004 |  |  |  | exp | -0.00258 | 0.00076 | -3.38 | 0.0007 |
|  |  |  |  | C. Total | 6860 | 2.840 | 0.0000 |  |  |  | ess[0] | 0.00344 | 0.00040 | 8.53 | <.0001 |
|  |  |  |  |  |  |  |  |  |  |  | (exp-0.66943)*ess[0] | 0.00014 | 0.00076 | 0.18 | 0.8539 |
|  |  |  |  |  |  |  |  |  |  |  |  |  |  |  |  |
| G | 6861 | 0.013 |  | Model | 3 | 0.030 | 0.0101 | 30.76 | <.0001 |  | Intercept | -0.00805 | 0.00070 | -11.51 | <.0001 |
|  |  |  |  | Error | 6857 | 2.241 | 0.0003 |  |  |  | exp | -0.00088 | 0.00069 | -1.28 | 0.2007 |
|  |  |  |  | C. Total | 6860 | 2.272 | 0.0000 |  |  |  | ess[0] | 0.00270 | 0.00036 | 7.46 | <.0001 |
|  |  |  |  |  |  |  |  |  |  |  | (exp-0.66943)*ess[0] | -0.00162 | 0.00069 | -2.36 | 0.0184 |
|  |  |  |  |  |  |  |  |  |  |  |  |  |  |  |  |
| T | 6861 | 0.025 |  | Model | 3 | 0.085 | 0.0285 | 57.83 | <.0001 |  | Intercept | 0.00452 | 0.00086 | 5.26 | <.0001 |
|  |  |  |  | Error | 6857 | 3.379 | 0.0005 |  |  |  | exp | -0.00696 | 0.00084 | -8.26 | <.0001 |
|  |  |  |  | C. Total | 6860 | 3.465 | 0.0000 |  |  |  | ess[0] | 0.00027 | 0.00044 | 0.6 | 0.5468 |
|  |  |  |  |  |  |  |  |  |  |  | (exp-0.66943)*ess[0] | -0.00142 | 0.00084 | -1.69 | 0.0909 |

Abbreviations: Nuc: Nucleotide; Obs: Observations; DF: Degrees of Freedom; exp: expression; ess: essential (ess[0]: non-essential)
