# Supplementary material for: DNA Sequences Shaped by Selection for Stability
Source: PLoS Genet. 2006 Feb 24;2(2):e22. doi: 10.1371/journal.pgen.0020022 (PMC1378130; doi:10.1371/journal.pgen.0020022)
Supplement: Table S3 — The binary response variable is codon choice (heterogeneous codon vs. homogeneous codon); the explanatory variable is the number of critical nucleotides following. In 11 out of 12 tests, the probability for a heterogeneous codon increased significantly with increasing numbers of critical nucleotides following. (187 KB DOC) [file pgen.0020022.st003.doc]

Table S3: Logistic Regression

| ***E. coli*** | |  |  |  |  |  |  |  |  |  |  |  |  |  |
| --- | --- | --- | --- | --- | --- | --- | --- | --- | --- | --- | --- | --- | --- | --- |
|  |  |  |  | Whole Model Test | |  |  |  |  | Parameter Estimates | |  |  |  |
| Nuc | Obs | R^2^ |  | Model | -Log P | DF | ChiSquare | Prob>ChiSq | | Term | Estimate | Std Error | ChiSquare | Prob>ChiSq |
| A | 59888 | 0.0011 |  | Difference | 35.9 | 1 | 71.8 | <.0001 |  | Intercept | -1.222 | 0.011 | 12636 | 0 |
|  |  |  |  | Full | 32620.6 |  |  |  |  | no_following | 0.096 | 0.011 | 73.85 | <.0001 |
|  |  |  |  | Reduced | 32656.5 |  |  |  |  | For log odds of HETERO/HOMO | | |  |  |
|  |  |  |  |  |  |  |  |  |  |  |  |  |  |  |
| C | 60170 | 0.0164 |  | Difference | 371.4 | 1 | 742.7 | <.0001 |  | Intercept | 1.790 | 0.013 | 18157 | 0 |
|  |  |  |  | Full | 22240.0 |  |  |  |  | no_following | 0.772 | 0.032 | 577.88 | <.0001 |
|  |  |  |  | Reduced | 22611.3 |  |  |  |  | For log odds of HETERO/HOMO | | |  |  |
|  |  |  |  |  |  |  |  |  |  |  |  |  |  |  |
| G | 100147 | 0.0194 |  | Difference | 825.2 | 1 | 1650.5 | 0 |  | Intercept | 1.507 | 0.010 | 22677 | 0 |
|  |  |  |  | Full | 41673.9 |  |  |  |  | no_following | 0.638 | 0.017 | 1363.1 | <.0001 |
|  |  |  |  | Reduced | 42499.1 |  |  |  |  | For log odds of HETERO/HOMO | | |  |  |
|  |  |  |  |  |  |  |  |  |  |  |  |  |  |  |
| T | 52988 | 0.0035 |  | Difference | 125.9 | 1 | 251.7 | <.0001 |  | Intercept | -0.355 | 0.010 | 1387.4 | <.0001 |
|  |  |  |  | Full | 36022.6 |  |  |  |  | no_following | 0.208 | 0.013 | 249.34 | <.0001 |
|  |  |  |  | Reduced | 36148.4 |  |  |  |  | For log odds of HETERO/HOMO | | |  |  |
|  |  |  |  |  |  |  |  |  |  |  |  |  |  |  |
|  |  |  |  |  |  |  |  |  |  |  |  |  |  |  |
| ***S. cerevisiae*** | |  |  |  |  |  |  |  |  |  |  |  |  |  |
|  |  |  |  | Whole Model Test | |  |  |  |  | Parameter Estimates | |  |  |  |
| Nuc | Obs | R^2^ |  | Model | -Log P | DF | ChiSquare | Prob>ChiSq | | Term | Estimate | Std Error | ChiSquare | Prob>ChiSq |
| A | 170799 | 0.0012 |  | Difference | 136.8 | 1 | 273.6 | <.0001 |  | Intercept | -0.360 | 0.006 | 3931.2 | 0 |
|  |  |  |  | Full | 116214.6 |  |  |  |  | no_following | 0.082 | 0.005 | 274.05 | <.0001 |
|  |  |  |  | Reduced | 116351.4 |  |  |  |  | For log odds of HETERO/HOMO | | |  |  |
|  |  |  |  |  |  |  |  |  |  |  |  |  |  |  |
| C | 99470 | 0.0032 |  | Difference | 138.5 | 1 | 277.0 | <.0001 |  | Intercept | 1.627 | 0.009 | 30524 | 0 |
|  |  |  |  | Full | 42998.3 |  |  |  |  | no_following | 0.279 | 0.018 | 246.77 | <.0001 |
|  |  |  |  | Reduced | 43136.8 |  |  |  |  | For log odds of HETERO/HOMO | | |  |  |
|  |  |  |  |  |  |  |  |  |  |  |  |  |  |  |
| G | 116360 | 0.0065 |  | Difference | 278.8 | 1 | 557.6 | <.0001 |  | Intercept | 1.876 | 0.010 | 34390 | 0 |
|  |  |  |  | Full | 42366.3 |  |  |  |  | no_following | 0.378 | 0.017 | 492.79 | <.0001 |
|  |  |  |  | Reduced | 42645.2 |  |  |  |  | For log odds of HETERO/HOMO | | |  |  |
|  |  |  |  |  |  |  |  |  |  |  |  |  |  |  |
| T | 101493 | 0.001 |  | Difference | 71.0 | 1 | 142.0 | <.0001 |  | Intercept | -0.324 | 0.007 | 2116.3 | 0 |
|  |  |  |  | Full | 68655.4 |  |  |  |  | no_following | -0.100 | 0.008 | 139.54 | <.0001 |
|  |  |  |  | Reduced | 68726.4 |  |  |  |  | For log odds of HETERO/HOMO | | |  |  |
|  |  |  |  |  |  |  |  |  |  |  |  |  |  |  |
|  |  |  |  |  |  |  |  |  |  |  |  |  |  |  |
| ***C. elegans*** | |  |  |  |  |  |  |  |  |  |  |  |  |  |
|  |  |  |  | Whole Model Test | |  |  |  |  | Parameter Estimates | |  |  |  |
| Nuc | Obs | R^2^ |  | Model | -Log P | DF | ChiSquare | Prob>ChiSq | | Term | Estimate | Std Error | ChiSquare | Prob>ChiSq |
| A | 121557 | 0.007 |  | Difference | 586.4 | 1 | 1172.9 | <.0001 |  | Intercept | -0.194 | 0.007 | 835.79 | <.0001 |
|  |  |  |  | Full | 83581.8 |  |  |  |  | no_following | 0.222 | 0.007 | 1140.6 | <.0001 |
|  |  |  |  | Reduced | 84168.3 |  |  |  |  | For log odds of HETERO/HOMO | | |  |  |
|  |  |  |  |  |  |  |  |  |  |  |  |  |  |  |
| C | 97083 | 0.0054 |  | Difference | 139.6 | 1 | 279.2 | <.0001 |  | Intercept | 2.407 | 0.013 | 33880 | 0 |
|  |  |  |  | Full | 25929.2 |  |  |  |  | no_following | 0.369 | 0.024 | 240.11 | <.0001 |
|  |  |  |  | Reduced | 26068.9 |  |  |  |  | For log odds of HETERO/HOMO | | |  |  |
|  |  |  |  |  |  |  |  |  |  |  |  |  |  |  |
| G | 116422 | 0.0184 |  | Difference | 498.9 | 1 | 997.8 | <.0001 |  | Intercept | 2.492 | 0.013 | 34741 | 0 |
|  |  |  |  | Full | 26631.8 |  |  |  |  | no_following | 0.705 | 0.025 | 781.72 | <.0001 |
|  |  |  |  | Reduced | 27130.7 |  |  |  |  | For log odds of HETERO/HOMO | | |  |  |
|  |  |  |  |  |  |  |  |  |  |  |  |  |  |  |
| T | 78994 | 0.0001 |  | Difference | 3.7 | 1 | 7.3 | 0.0069 |  | Intercept | 0.377 | 0.008 | 2238.7 | 0 |
|  |  |  |  | Full | 53309.9 |  |  |  |  | no_following | 0.030 | 0.011 | 7.28 | 0.007 |
|  |  |  |  | Reduced | 53313.5 |  |  |  |  | For log odds of HETERO/HOMO | | |  |  |
|  |  |  |  |  |  |  |  |  |  |  |  |  |  |  |

Abbreviations: Nuc: Nucleotide; Obs: Observations; R2: R-Square; -Log P: - Log Likelihood; DF: Degrees of Freedom ; HETERO/HOMO: heterogeneous codon vs. homogeneous codon; no_following: number of critical nucleotides following the focal codon.
